# Reliability and Validity of the Persian Version of the Fatigue Severity Scale in Idiopathic Parkinson's Disease Patients

**DOI:** 10.1155/2013/935429

**Published:** 2013-09-05

**Authors:** Seyed-Mohammad Fereshtehnejad, Hasti Hadizadeh, Farzaneh Farhadi, Gholam Ali Shahidi, Ahmad Delbari, Johan Lökk

**Affiliations:** ^1^Division of Clinical Geriatrics, Department of Neurobiology, Care Sciences, and Society (NVS), Karolinska Institutet, 14186 Stockholm, Sweden; ^2^Firoozgar Clinical Research Development Center (FCRDC), Firoozgar Hospital, Iran University of Medical Sciences, Tehran 15937-48711, Iran; ^3^Medical Student Research Committee (MSRC), Faculty of Medicine, Iran University of Medical Sciences, Tehran 14496-14535, Iran; ^4^Movement Disorders Clinic, Department of Neurology, Faculty of Medicine, Iran University of Medical Sciences, Tehran 14496-14535, Iran; ^5^Department of Geriatrics Medicine, Karolinska University Hospital, 14186 Stockholm, Sweden

## Abstract

As one of the most frequent symptoms, measurement of fatigue is an issue of interest in Parkinson's disease (PD). The fatigue severity scale (FSS) is one of the recommended questionnaires for this purpose. The aim of our study was to evaluate psychometric properties of the Persian version of the FSS (FSS-Per) to assess fatigue in PD patients. Ninety nondemented idiopathic Parkinson's disease (IPD) patients were consecutively recruited from an outpatient referral movement disorder clinic. In addition to the disease severity scales, the FSS-Per was used for fatigue measurement. The internal consistency coefficient was larger than 0.8 for all of the items with a total Cronbach's alpha of 0.96 (95% CI: 0.95–0.97). The FSS-Per score correlated with the UPDRS score (*r* = 0.55, *P* < 0.001) and the “Hoehn and Yahr” (HY) stage (*r* = 0.48, *P* < 0.001). The total score of the FSS-Per significantly discriminated IPD patients with more severe disability (HY stage > 2) versus those with less severe disease (HY stage ≤2) (AUC = 0.81 (95% CI: 0.72–0.90)). The FSS-Per fulfilled a high internal consistency and construct validity to measure the severity of fatigue in Iranian IPD patients. These acceptable psychometric properties were reproducible in subgroups of IPD patients regarding different levels of education, disease severity, sex and age groups.

## 1. Introduction

Fatigue is one of the most disabling nonmotor symptoms of Parkinson's disease (PD) [1]. It has been defined as a feeling of abnormal and overwhelming tiredness and shortage of energy, which is distinct from normal tiredness both in quality and quantity [2]. Because of its multidimensionality and lack of a universal definition, there are a large number of questionnaires measuring different aspects of fatigue [3]. However, there is no single multidimensional questionnaire, validated in patients with PD [3]. Based on a review done by the International Movement Disorders Society (IMDS), only a few scales are recommended for measuring fatigue severity in PD, one of which is the fatigue severity scale (FSS) [4].

The FSS is a self-report, one-dimensional scale, which was primarily developed in 1989 for patients with multiple sclerosis [5]. This scale does not specifically measure cognitive fatigue [6] and contains nine brief items, each of which is graded from 1 (strong disagreement) to 7 (strong agreement) [5]. Although many studies have previously shown the clinimetric properties of the FSS in different chronic diseases [5, 7–11], there are few validation reports in PD. On the other hand, even though the FSS has been validated in different languages, there is no study to use the Persian version of the scale to assess fatigue in PD. Our study aimed to evaluate the reliability and validity of the Persian version of the FSS (FSS-Per) as an instrument to assess fatigue in PD patients.

## 2. Methods

### 2.1. Study Setting

Between October 2011 and September 2012, a total number of 90 idiopathic Parkinson's disease (IPD) patients were consecutively recruited from an outpatient referral Movement Disorder Clinic in Tehran, Iran. This cross-sectional study was a collaborative project between Karolinska Institutet, Stockholm, Sweden, and Iran University of Medical Sciences (IUMS), Tehran, Iran.

### 2.2. Ethical Issues

The study protocol was approved by the Ethics Committee of the Firoozgar Clinical Research Development Center (FCRDC) (affiliated to Iran University of Medical Sciences) in Tehran, Iran. Each participant was informed about the aims and objectives of the study before participation, and the completion of the questionnaire was voluntary. Furthermore, the identity of research participants was protected, since the data files were anonymous.

### 2.3. Patients' Recruitment and Assessment

Patients were eligible if diagnosis of IPD was confirmed using the United Kingdom (UK) brain bank criteria [12] after a complete clinical examination done by one neurologist specialized in movement disorders. Recruited patients were required to be 35 years or older, and those with moderate to severe dementia with the Mini-Mental State Examination (MMSE) [13] of <24 were excluded from the study. In addition, any patient with atypical parkinsonian syndromes such as multiple system atrophy (MSA), vascular parkinsonism, drug-induced parkinsonism, and progressive supranuclear palsy (PSP) was excluded.

Data collection was performed using a series of questionnaires to assess various aspects of the disease during the interview session and clinical examination. Basic demographic information consisted of baseline variables, educational status, and comorbidities. PD-related characteristics including disease duration (time passed from diagnosis), measures of disease severity such as Hoehn and Yahr stage [14], Schwab and England activity of daily living (ADL) scale [15], the Unified Parkinson's Disease Rating Scale (UPDRS) score [16], and levodopa cumulative daily dosage were also recorded.

The Hoehn and Yahr staging [14] is a widely used clinical rating scale, supplanted by the UPDRS, which evaluates the severity of PD based on motor functional disability and clinical findings consisting of 5 stages. Stage 0 indicates no visible symptoms of PD, and 5 shows symptoms on both sides of the body indicating the PD patients who are unable to walk. Therefore, a higher stage shows greater levels of functional disability [14]. The Schwab and England scale [15] is another global scoring system for assessing a PD patient's ability to perform daily activities in terms of speed and independence through a percentage figure, where 100% indicates total independence, falling to 0%, which indicates a state of complete dependence in bed-ridden individuals. Therefore, higher scores show greater level of independence [15]. As the most commonly used scale in the clinical study of PD [17], we also used the UPDRS to assess the severity of Parkinson's disease in different aspects including nonmotor symptoms (part I), motor symptoms (part II), motor examination (part III), and drug complications (part IV). The UPDRS is scored from a total of 147 points where higher scores reflect worsening disability [16].

After clinical assessment, patients answered the Persian version of the fatigue severity scale (FSS-Per) questionnaire supervised by a group of trained medical students for clarification and to avoid missing information. 

### 2.4. Fatigue Severity Scale (FSS) Questionnaire

The Fatigue Severity Scale (FSS) is a self-report instrument assessing the physical aspects of fatigue and their impact on the patient's daily function in a variety of medical and neurologic disorders. It evaluates the impact of fatigue on motivation, exercise, physical functioning, carrying out duties and responsibilities, and interfering with work, family, or social life. It contains nine items in the format of brief and understandable statements. Patients were asked to rate their level of fatigue during the past week using a seven-grade Likert scale. The rating scores range from 1 to 7 for each statement; however, only the respective ends of the scale are defined where a low value of 1 indicates “*completely disagree*” with the statement and a high value of 7 indicates “*completely agree*” or the most severe fatigue. The total FSS score represents the mean score of the nine items ranging between 1 and 7 where the higher scores indicate more severe fatigue [5].

The FSS questionnaire was previously translated into Persian language and showed acceptable validity and reliability among patients with multiple sclerosis [18]. In this project, we used this Persian-translated version of the FSS (FSS-Per) in IPD patients.

### 2.5. Statistical Analysis

#### 2.5.1. Description

Data were analyzed by SPSS software version 17.0 (Chicago, IL, USA). In order to describe continuous and qualitative variables, mean (standard deviation (SD)) and frequency (percentage) were used, respectively. The minimum, maximum, and coefficient of variation (CV) were also reported for each of the items in FSS-Per questionnaire. The principal components analysis (factor analysis) was applied to explore the best fitted factors with an eigenvalue of >1 to detect the structure of the FSS. 

#### 2.5.2. Reliability

Internal consistency was checked using the Spearman correlation statistic where mean score of each item was correlated with the sum of the FSS-Per score. Furthermore, Cronbach's alpha intraclass coefficient and the 95% confidence interval (CI) of the point estimations were calculated for the whole questionnaire and within different subgroups of IPD patients. 

#### 2.5.3. Validity

Spearman correlation was used to evaluate the convergent validity of the total score of the FSS-Per questionnaire in association with the baseline and PD-related variables. To check the construct validity, the receiver operating characteristics (ROC) curve statistic was applied to assess whether the total score of the FSS-Per could discriminate IPD patients with more severe disease. For this purpose, IPD patients were divided into two groups: more severe disability (Hoehn and Yahr stage >2) versus less severe disease (Hoehn and Yahr stage ≤2). The area under curve (AUC) and its corresponding 95% CI were calculated. Thereafter, a cut-off value was selected where the best diagnostic indices (sensitivity and specificity) were met. 

In all analytical procedures, a two-sided *P* value <0.05 was considered as the statistical significant level to reject the beyond H0 hypothesis.

## 3. Results

### 3.1. Baseline Characteristics

Study samples consisted of 62 (68.9%) male and 28 (31.1%) female patients with the mean age of 62.0 (SD = 10.7) years ranging between 38 and 91 years and the median PD duration of 5.0 years. The majority of patients were in the mild to moderate stage of PD where 70% (63 out of 90) had the Hoehn and Yahr stage of ≤2 with the mean UPDRS score of 32.5 (SD = 18.3). Other baseline, sociodemographic, and clinical characteristics of the patients are shown in Table 1.

### 3.2. FSS-Per Structural Characteristics

As it is shown in Table 2, the mean of the total FSS score was 4.4 (SD = 2.0) ranging between 1 and 7 in Iranian PD patients. The 4th item on “*the interfering of fatigue with physical functioning*” showed the highest score (4.9 (SD = 2.1)), whereas the last item on “*the interfering of fatigue with work, family and social life*” had the lowest severity score (3.8 (SD = 2.3)). The largest and smallest coefficient of variation (CV) was also observed in items 9 (60.5%) and 4 (42.9%), respectively.

In the principal components analysis, the first explored component of the FSS extracted 76.7% of the common variance with an eigenvalue of 6.9. However, the eigenvalues dramatically dropped for the next factors and failed to attain value above 1.0. The second factor had an eigenvalue of 0.53 indicating that a one-factor structure is the most appropriate one for the FSS-Per in IPD patients.

### 3.3. FSS-Per Reliability

As shown in Table 2, the Spearman Rho was larger than 0.8 for all of the items (all *P* values <0.001). The highest correlation coefficients were calculated for the 5th (*r* = 0.935) and 6th (*r* = 0.932) items, respectively, while the 1st (*r* = 0.803) and 3rd (*r* = 0.827) items showed the lowest internal consistency.

**Figure 1:**
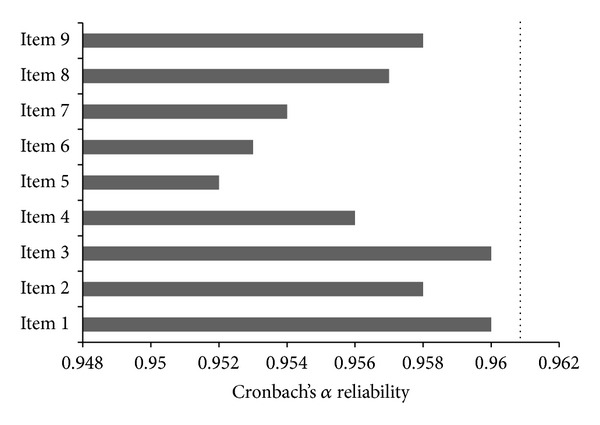
Changes in the reliability index (Cronbach's *α*) after deletion of each single item of the FSS questionnaire in Iranian Parkinson's disease patients (*n* = 90) (the dotted line represents total Cronbach's *α* of the questionnaire (0.961)).

The whole FSS-Per questionnaire had statistically significant reliability with the Cronbach's alpha coefficient of 0.961 (95% CI: 0.948–0.972, *P* < 0.001).  Figure 1 illustrates the predicted changes in the reliability index of the FSS-Per questionnaire if any of the single items were deleted. In general, the combination of all 9 items showed the highest reliability where the deletion of none of the items increased the total Cronbach's alpha coefficient. Moreover, the exclusion of items 5 and 6 demonstrated the highest decrease in the reliability of the FSS-Per questionnaire, while the absence of the items 1 and 3 had the smallest effect on the Cronbach's alpha coefficient. Further analysis resulted in Cronbach's alpha coefficient of larger than 0.9 in all of the subgroups regarding age, sex, educational level, and PD severity (all *P* value <0.001). However, based on the subgroups' specific 95% CIs, the questionnaire had significantly higher Cronbach's alpha among the males (0.966 (95% CI: 0.952–0.978) versus 0.929 (95% CI: 0.882–0.963)) and higher educated PD patients (0.980 (95% CI: 0.969–0.989) versus 0.942 (95% CI: 0.916–0.963)).

### 3.4. FSS-Per Validity

As shown in Table 3, PD duration and severity were significantly correlated with the FSS-Per score. Besides all of the domains of the UPDRS, its total score was directly correlated with fatigue severity (*r* = 0.548, *P* < 0.001). The “Hoehn and Yahr” stage (*r* = 0.478, *P* < 0.001) and the “Schwab and England” ADL scale (*r* = −0.487, *P* < 0.001) were also significantly correlated with the total score of the FSS-Per questionnaire.

**Figure 2:**
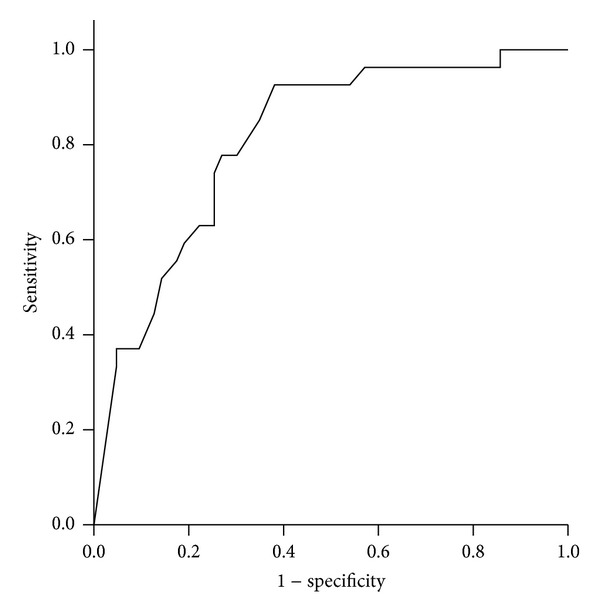
Receiver operating characteristics (ROC) curve for the total score of the FSS-Per questionnaire to discriminate Parkinson's disease patients with more severe disability with Hoehn and Yahr stage >2 (area under curve (AUC) = 0.81, *P* < 0.001).

Figure 2 illustrates the result of the ROC analysis where the total score of the FSS-Per questionnaire has significant value to discriminate IPD patients with more severe disability (Hoehn and Yahr stage >2) versus those with less severe disease (Hoehn and Yahr stage ≤2) (AUC = 0.81 (95% CI: 0.72–0.90); *P* < 0.001). The cut-off value of 4.5 for the mean of the total FSS score showed the best diagnostic value for this discrimination with 92.6% sensitivity and 61.9% specificity.

## 4. Discussion

Our study is one of the first attempts to use the Persian-translated FSS questionnaire in Iranian Parkinson's disease patients in order to evaluate their fatigue. For this purpose, trans-cultural validation of the instrument is a matter of the utmost importance. Based on the reliability analysis, the FSS-Per showed an acceptable internal consistency (correlation *r* > 0.75 in all items) and a very high intraclass correlation (overall Cronbach's *α* = 0.96). In addition, the validity of the FSS-Per was approved in IPD patients by means of correlation with the measures of disease severity and discriminative ability to detect more severe disability. 

Table 4 summarizes the few similar reports to evaluate the psychometric properties of the FSS in PD patients with different native languages. Similar to our study, a very high reliability has been shown in all of these studies with a Cronbach's alpha coefficient ranging from 0.91 to 0.95 [19–21]. Hagell et al. [19] performed a psychometric study on the Swedish version of the FSS in 118 consecutive PD patients with all five Hoehn and Yahr stages of disease severity. They demonstrated that the Swedish-translated FSS had an excellent reliability based on Cronbach's alpha of 0.94 [19]. More similar to our findings, the Brazilian-Portuguese translation of the FSS had shown a Cronbach's alpha of 0.95 in a sample of 30 PD patients [21]. In another study done by Grace et al. [20], an acceptable internal consistency of the English version of the FSS was demonstrated by a split half reliability of 0.86 and 0.91 in 50 PD patients. They also showed the interitem correlations between 0.27 and 0.78 for the FSS in PD patients [20]. We observed even higher internal consistency in the correlations between each single item and sum of the scores of the entire FSS ranged from 0.76 to 0.92. However, the first item on the patients' motivation showed the lowest coherence in the item-total correlation in our study on PD patients. This finding is in line with a previous report on the Norwegian-translated version of the FSS in general population where item 1 showed the lowest average correlation with the rest of the items and the total score [22]. Using the Rasch statistical method, Hagell et al. [19] also found that item 1 did not meet unidimensionality criteria with other items in the FSS. Nevertheless, exploratory factor analysis supported the unidimensionality of the entire scale in PD [19], which is similar to our report on the FSS-Per.

Regarding the construct validity of the FSS-Per, our data showed moderate to strong correlation between the total score of the FSS and indices of PD severity such as Hoehn and Yahr Stage, Schwab and England ADL Scale, and total UPDRS score. Recently, Valderramas et al. [21] and Herlofson and Larsen [23] showed significant correlations between the total score of the FSS and Hoehn and Yahr and UPDRS scores in Brazilian and Norwegian IPD patients, respectively. Similar to our report, both of these previous studies showed a higher correlation with the total UPDRS score where the nonmotor symptoms are also considered to estimate PD severity. This attributes to the important role of the nonmotor symptoms in fatigue complaints among PD patients [24].

In addition to correlation assessments, we also performed ROC analysis to evaluate the validity of the FSS scores in IPD patients. Our findings indicated that the entire FSS score significantly discriminates between more severe disability and the IPD patients with less severe stages in term of Hoehn and Yahr staging. This finding provides more evidence to support the validity of the FSS in IPD patients. Although this discriminative ability of the FSS has been previously shown to distinguish between diseased and normal population [11, 25–27], our study is one of the first reports to show this discrimination between more and less severe PD using the FSS scores. 

In our study, further subgroup investigation demonstrated that the FSS-Per could be used as a reliable fatigue-specific scale in IPD patients with different age group, sex, educational level, and disease severity. Statistical analysis showed acceptable reliability indices even among the older and less educated IPD patients. In the same way but based on different statistical methods, Hagell et al. [19] yielded significant item calibration for the FSS in subsets of PD patients regarding sex and age groups.

Our study was designed as a cross-sectional project. This lack of temporality leads to some limitations. It was not possible to evaluate the validity of the FSS-Per to measure changes in fatigue during the course of PD. Although our study covered IPD patients with different level of disease severity, there was skewness toward less severe stages, and enrollment criteria excluded patients with clinically significant cognitive impairment. This selection bias limits the generalisability of results; however, the main objective of our study was to assess the psychometric properties of the FSS-Per and not to provide a representative picture of fatigue in IPD.

In conclusion, our study demonstrates that the Persian-translated version of the FSS fulfills the criteria of a reliable and valid assessment tool to rate the severity of fatigue in IPD patients. This scale showed good psychometric properties in IPD patients with different levels of education, disease severity, and sex and age groups. The high internal consistency and construct validity support the application of the FSS as an easy-administered tool to evaluate fatigue in IPD patients.

## Figures and Tables

**Table 1:** Baseline, clinical, and sociodemographic characteristics of the Parkinson's disease patients (*n* = 90).

Characteristics	Value
Age (yr)	
Mean (SD)	62.0 (10.7)
Gender number (%)	
Female	28 (31.1)
Male	62 (68.9)
Level of education number (%)	
Illiterate	4 (4.5)
Primary and/or secondary	22 (24.7)
High school/diploma	28 (31.5)
College and/or university	35 (39.3)
Duration of disease (yr)	
Mean (SD)	6.0 (4.8)
Comorbidities number (%)	
Depression	22 (24.4)
Hypertension	13 (14.4)
Cardiovascular disease	13 (14.4)
Diabetes	13 (14.4)
Osteoarthritis	8 (8.9)
UPDRS score	
Mean (SD)	
Part I-*mental *	1.9 (2.3)
Part II-ADL	11.5 (7.3)
Part III-motor	15.4 (9.5)
Part IV-complications	3.7 (2.8)
Total	**32.5** (**18.3**)
Hoehn and Yahr stage	
Mean (SD)	1.9 (0.9)
Schwab and England activities of daily living score (%)	
Mean (SD)	81.9 (16.7)
Daily levodopa dose (mg)	
Mean (SD)	809 (484)

**Table 2:** Descriptive characteristics and the Spearman correlation of each item for internal consistency of the FSS questionnaire in Iranian Parkinson's disease patients (*n* = 90).

Item	Mean	SD.	CV.	Spearman* Rho *	Corrected item/total correlation
Item 1 *My motivation is lower when I am fatigued *	4.6	2.3	50	0.803	0.760
Item 2 *Exercise brings on my fatigue *	4.6	2.2	47.8	0.865	0.816
Item 3 *I am easily fatigued *	4.2	2.4	57.1	0.827	0.760
Item 4 *Fatigue interferes with my physical functioning *	4.9	2.1	42.9	0.897	0.852
Item 5 *Fatigue causes frequent problems for me *	4.1	2.4	58.5	0.935	0.922
Item 6 *My fatigue prevents sustained physical functioning *	4.4	2.3	52.3	0.932	0.921
Item 7 *Fatigue interferes with carrying out certain duties and responsibilities *	4.2	2.2	52.4	0.910	0.893
Item 8 *Fatigue is amongst my three most disabling symptoms *	4.4	2.4	54.5	0.851	0.830
Item 9 *Fatigue interferes with my work, family, or social life *	3.8	2.3	60.5	0.848	0.799
Total score	4.4	2.0	45.4	—	—

SD.: standard deviation; CV.: coefficient of variation.

**Table 3:** The Spearman correlation to evaluate the convergent validity of the FSS questionnaire in association with the baseline and disease-related variables in Iranian Parkinson's disease patients (*n* = 90).

Scale/variable	Spearman* Rho *	*P* value
Age	−0.070	0.512
Duration of disease	0.284	0.007*
UPDRS Score		
Part I-*mental *	0.264	0.012*
Part II-ADL	0.519	<0.001*
Part III-motor	0.521	<0.001*
Part IV-complications	0.231	0.029*
Total	0.548	<0.001*
Hoehn and Yahr stage	0.478	<0.001*
Schwab and England ADL scale	−0.487	<0.001*
Daily levodopa dose	0.128	0.233

*Statistical significant correlation (*P* < 0.05).

**Table 4:** Trans-cultural reliability and validity of the different language versions of the FSS questionnaire in Parkinson's disease patients.

Study group	Year	Language	Reliability		Validity	
			Internal consistency *(Spearman R) *	Internal consistency* (Cronbach's α*)	Correlation coefficient *(UPDRS) *	Correlation coefficient *(Hoehn and Yahr) *
Hagell et al. [19]	2006	Swedish	—	0.94	—	—
Grace et al. [20]	2007	English	0.44–0.78	0.91	—	—
Valderramas et al. [21]	2012	Brazilian-Portuguese	—	0.95	0.45	0.40
Current study	2013	Persian	0.76–0.92	0.96	0.55	0.48
